# Android mobile-platform-based image reconstruction for photoacoustic tomography

**DOI:** 10.1117/1.JBO.28.4.046009

**Published:** 2023-04-27

**Authors:** Xie Hui, Praveenbalaji Rajendran, Muhamad Ar Iskandar Zulkifli, Tong Ling, Manojit Pramanik

**Affiliations:** a Nanyang Technological University, School of Chemistry, Chemical Engineering and Biotechnology, Singapore; b Stanford University, Department of Radiation Oncology, Stanford, California, United States; c Iowa State University, Department of Electrical and Computer Engineering, Ames, Iowa, United States

**Keywords:** photoacoustic tomography, image reconstruction, Android application, mobile system, app development

## Abstract

**Significance:**

In photoacoustic tomography (PAT), numerous reconstruction algorithms have been utilized to recover initial pressure rise distribution from the acquired pressure waves. In practice, most of these reconstructions are carried out on a desktop/workstation and the mobile-based reconstructions are far-flung. In recent years, mobile phones are becoming so ubiquitous, and most of them encompass a higher computing ability. Hence, realizing PAT image reconstruction on a mobile platform is intrinsic, and it will enhance the adaptability of PAT systems with point-of-care applications.

**Aim:**

To implement PAT image reconstruction in Android-based mobile platforms.

**Approach:**

For implementing PAT image reconstruction in Android-based mobile platforms, we proposed an Android-based application using Python to perform beamforming process in Android phones.

**Results:**

The performance of the developed application was analyzed on different mobile platforms using both simulated and experimental datasets. The results demonstrate that the developed algorithm can accomplish the image reconstruction of *in vivo* small animal brain dataset in 2.4 s. Furthermore, the developed application reconstructs PAT images with comparable speed and no loss of image quality compared to that on a laptop. Employing a two-fold downsampling procedure could serve as a viable solution for reducing the time needed for beamforming while preserving image quality with minimal degradation.

**Conclusions:**

We proposed an Android-based application that achieves image reconstruction on cheap, small, and universally available phones instead of relatively bulky expensive desktop computers/laptops/workstations. A beamforming speed of 2.4 s is achieved without hampering the quality of the reconstructed image.

## 1 Introduction

Photoacoustic tomography (PAT)/photoacoustic computed tomography (PACT) is a hybrid imaging method that enables deep tissue imaging with a high spatial resolution by combining optical illumination with ultrasound detection.^1^^–^^4^ Over the last decade, PAT has shown great potential in preclinical and clinical applications due to its advantages, such as scalable resolution, higher imaging depth, and high contrast.^5^^–^^12^ PAT is based on the photoacoustic (PA) effect, where the PA waves are generated when incident laser energy is absorbed by the chromophores, such as oxy- and deoxyhemoglobin. In PAT, nanosecond laser pulses are employed to illuminate the tissue, leading to energy absorption and local temperature rise. This gives rise to pressure waves (PA), which are propagated out to the tissue boundary in the form of ultrasound waves (also known as PA waves). These PA waves are acquired using an ultrasound transducer (UST) at the tissue boundary. Then, they are converted into an internal absorption map (or to be precise initial pressure rise map) of the tissue with the aid of different reconstruction algorithms.

In PAT/PACT, circular scanning geometry in orthogonal mode is most widely used for deep tissue imaging,^13^ where a single-element ultrasound transducer (SUT) is employed to rotate around the sample in a full circle to acquire the PA signals at different locations. The acquired PA signals are then reconstructed into cross-sectional PAT images utilizing various reconstruction algorithms.^14^^–^^19^ Conventionally, SUT takes several minutes to acquire the data, leading to longer data acquisition (DAQ) time. Hence, array transducers such as linear array transducers,^20^^,^^21^ semi-circular transducers,^22^ and circular array transducers^23^ are used to improve imaging speed. The reconstruction techniques employed for reconstructing the acquired PA signals by these transducers remain the same as that of the SUT-based PAT systems. In comparison with the SUT, employing a circular array transducer with 128 or 256 elements reduces the DAQ time as it can simultaneously capture multiple time-resolved PA signals. However, the circular array transducers are custom-made and require complex back-end parallel signal processing.^24^ Hence, SUTs are preferred due to their easy availability and low cost. In this work, we focused on the SUT-based reconstruction algorithm, though the developed Android-based application can be easily applied to even the data from array transducers.

**Fig. 1:**
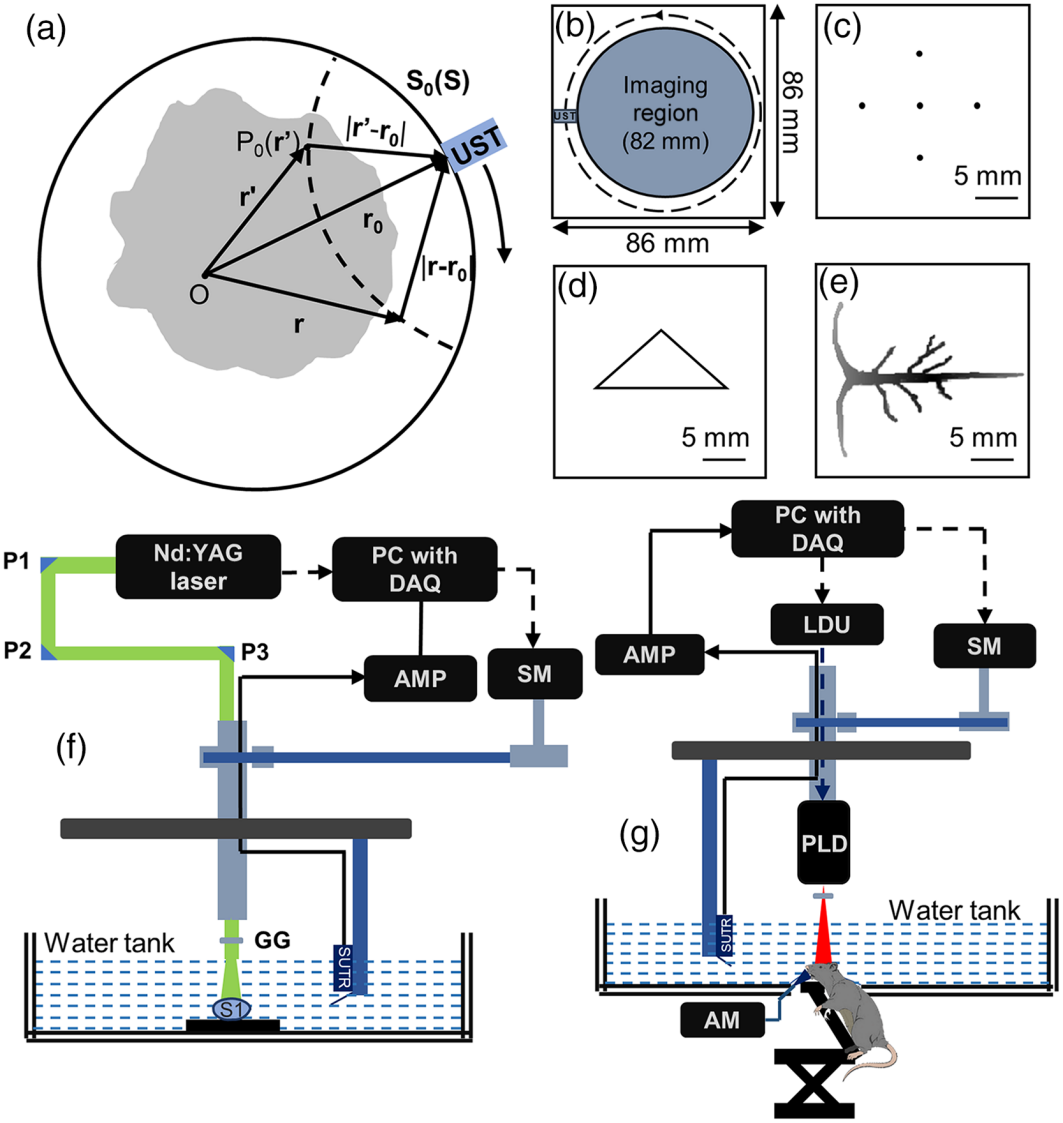
(a) Schematic diagram for backprojection process. (b) Simulation geometry used in MATLAB k-wave toolbox. (c) Point source numerical phantom (five-point targets; a point is located at the center and surrounded by the other four points). (d) Triangle source numerical phantom. (e) Numerical phantom that mimics the brain vessel of rat. (f) Schematic diagram of the PAT system for experimental phantom imaging. (g) Schematic diagram of the PLD-PAT system for *in vivo* imaging. AM, anesthesia machine; AMP, amplifier; LDU, laser driving unit; SM, stepper motor; PC, personal computer; DAQ, data acquisition card; PLD; pulsed laser diode; and GG, ground glass. P1, P2, and P3 are uncoated prisms; S1 is the sample used for phantom imaging.

Different reconstruction algorithms have been proposed to generate the initial pressure rise map from the acquired PA data, such as simple delay-and-sum (DAS) beamformer, time reversal method, iterative image reconstruction, Fourier-transform-based reconstruction, model-based reconstruction approach, etc.^15^^,^^16^^,^^25^^,^^26^ Assuming that the local optical fluence is locally homogeneous, we can recover the initial pressure distribution $p_{0}$ inside tissue by measuring the pressures at the tissue surface $p(\overset{\to }{r_{0}},t)$, followed by mapping $p_{0}$ into the absorption coefficient.^27^ Many methods can be used to reconstruct $p_{0}$ from $p(\overset{\to }{r_{0}},t)$). Herein, we only focus on the simple back-projection method, in which the initial pressure rise can be defined as follows:^28^

$$p(\overset{\to }{r_{0}})=\int_{\Omega_{0}}b(\overset{\to }{r_{0}},t=\frac{\left|\overset{\to }{r}-\overset{\to }{r_{0}}\right|}{v_{s}})\frac{d\Omega_{0}}{\Omega_{0}},$$

where $\Omega_{0}$ refers to the solid angle subtended by the entire measurement surface $S_{0}$ with respect to the reconstruction point $\vec{r}$ inside $S_{0}$, whereas $d\Omega_{0}$ refers to the solid angle subtended by the detection element $dS_{0}$ with respect to the reconstruction point at $\vec{r}$ inside $S_{0}$. $d\Omega_{0}/\Omega_{0}$ is a factor that weighs how much the detection element $dS_{0}$ contributes to the reconstruction. $\Omega_{0}=2\pi$ for planner geometry and $\Omega_{0}=4\pi$ for spherical geometry [Fig. 1(a)]. $b(\vec{r_{0}},t)$ denotes the backprojection term, which is given as

$$b(\overset{\to }{r_{0}},t)=2p(\overset{\to }{r_{0}},t)-2ct\frac{\partial p(\overset{\to }{r_{0}},t)}{\partial t};$$

$b(\vec{r_{0}},t)$ is projected backward on a spherical surface centered at the position $\vec{r_{0}}$. In a circular scanning geometry, a simple DAS beamformer is usually employed to implement back-projection with $b(\overset{\to }{r_{0}},t)=p(\overset{\to }{r_{0}},t)$. Signals from different recording locations are backprojected, and all the projected values are added at every pixel in the reconstructed image.^29^ DAS beamformer takes a relatively long time to be implemented due to expensive computation, and it induces artifacts in the reconstructed image owing to considering a large aperture detector in practice as a point detector during the reconstruction process. Despite the above-mentioned drawbacks, DAS beamformer still enjoys great popularity for its simplicity and easy implementation.

Typically, these reconstructions are performed on a desktop/laptop/workstation owing to their computational power. At present, mobile phones also have reasonable computing resources with the kind of processors and memory (RAM) they are using. With the upgrade of mobile phones every year, they possess more and more advanced processors [even with graphics processing units (GPUs)] and can be applied to many different fields, especially in biomedical imaging fields.^30^^–^^32^ Mobile phone-based imaging, sensing, and diagnostics have been proposed for microscopy modalities, such as bright-field^33^ and fluorescence microscopy,^34^^–^^36^ as well as for ultrasound imaging. Different Metal API-based applications have been proposed to achieve US beamforming reconstruction.^30^^,^^37^ For example, real-time ultrasound imaging formation on an iPhone or iPad can be achieved by utilizing wireless transmission of raw channel data from the ultrasound probe to the iPhone and the processing power of the GPU inside the phone.^30^ Here, an iOS app was developed, and the code for beamforming and image reconstruction was performed using Metal, an application program interface developed by Apple that accelerated GPU processing. For a single-angle plane-wave transmission, real-time frame rates of 60 to 90 frames per second have been achieved. Besides, a commercial smartphone (Samsung Galaxy Note 2) could be employed to implement vein visualization.^38^ Based on the Wiener estimation method, the multispectral information from the veins was acquired and visualized in real-time using an ultrasound processing application developed on the iOS platform, and signal processing of raw ultrasound data was accomplished for image generation. In recent years, the application for ultrasound beamforming has been commercialized. Butterfly Network has launched a product called Butterfly iQ+, where a US probe and an iOS or Android app were utilized for imaging.^39^ With the single probe, four types of imaging modes (e.g., B mode, M mode, Color Doppler, and Power Doppler) and over 20 presets (e.g., lung imaging, cardiac imaging) can be selected. The product not only supports real-time imaging with high frame rate, optimized beamforming, and storage but also allows bedside ultrasound remotely and provides real-time guidance. However, previous research on utilizing mobile phones for image processing mainly focused on the matured imaging modalities, such as ultrasound imaging,^40^ but was rarely applied to photoacoustic imaging. By designing an application on a mobile phone for raw data processing, image reconstruction can be fulfilled anytime and anywhere as mobile phones are ubiquitous. Based on portable devices for image formation, point-of-care diagnosis can therefore be developed. Mobile phones have advanced processors that allow images reconstructed on mobile devices to have comparable quality and fast processing time compared to the images reconstructed on a computer.

In this work, we propose a mobile-platform-based (Android) DAS algorithm to reconstruct the PAT image on phones. This is the first work where image reconstruction is conducted on mobile phones without using computers. The algorithm was employed to reconstruct the simulated and experimental PAT data into PAT images, achieving comparable efficiency and image quality to that of the DAS algorithm employed on a laptop. The generation of simulated PA data was performed on the k-wave MATLAB toolbox.^41^ Experimental phantom and *in vivo* imaging performed on rat brain vasculature were also used to verify the performance.

## 2 Methods

### 2.1 Simulated Photoacoustic Datasets

To evaluate the performance of the developed application, three numerical datasets such as point targets, triangular shape, and vessel shape were generated using the k-Wave toolbox.^41^ Although in practice UST with a large active area is used, an ideal point detector was used for simulation. A circular scanning geometry with a scanning radius of 41 mm was used. The computational grid was 820×820 (0.1  mm/pixel) with a perfectly matched bounding layer [Fig. 1(b)]. An ideal detector (2.25 MHz central frequency and 70% nominal bandwidth) was utilized for acquiring the PA data at 800 uniformly distributed positions around the sample, with 1500 steps at each position and a sampling frequency of 25 MHz. The speed of sound considered was 1500  m/s and the medium chosen was acoustically homogeneous. The first numerical phantom consisted of five-point targets, as shown in Fig. 1(c). The distance between two adjacent points was 5 mm while the intermediate point was located at the scanning center. Figures 1(d) and 1(e) present the numerical phantom of the isosceles triangle and brain vasculature that mimics the venous sinuses of rat brain, respectively.

### 2.2 Experimental Phantom Imaging

For experimental phantom imaging, two types of phantoms were used for validating the performance of the developed application, namely a point source phantom that was made up of pencil leads and a triangular phantom made up of horsehair. As shown in Fig. 1(f), a ∼532-nm Q-switched Nd:YAG laser delivering 10 pulses per second with a pulse width of 5 ns was used as illumination source.^42^ An unfocused UST (Olympus NDT, V306-SU) with a central frequency of 2.25 MHz, active area of 13 mm, and fractional bandwidth of 70%, was used to scan the sample with a scanning time of 480 s. 4800 A-lines were obtained during the 8-min scanning process, and for each A-line, 1024 samples were captured with a sampling frequency of 25 MHz. Averaging was performed for every two A-lines so that the number of A-lines became 2400. The collected PA signals were amplified via a low signal noise amplifier (Olympus-NDT, 5072PR) and stored in a computer (IntelXeon, 3.7 GHz 64-bit processor, 16 GB RAM) using a DAQ card (GaGe, compuscope 4227).

### 2.3 *In Vivo* Photoacoustic Imaging

The schematic diagram of the pulsed laser diode (PLD)-based PA imaging system, PLD-PAT system^43^ that was used for *in vivo* imaging is shown in Fig. 1(g). The PLD produced ∼816  nm light with a pulse repetition rate of 2000 Hz and a pulse width of ∼107  nm. A laser driving unit (LDU) was used to control the PLD. The LDU comprises a water-cooling unit, a low voltage power supply of 12 V (Voltcraft, PPS-11810), a variable high voltage power supply (Elektro-Automatik, EAPS 8160-04T) that can control the laser power, and a function generator (RIGOL, DG1022) that can control the pulse repetition rate. The laser beam homogenized by an optical diffuser was expanded over an area of $∼20\;{\mathrm{cm}}^{2}$. The laser irradiated on the sample surface met the requirements of American National Standards Institute safety limit.^44^ Single-element unfocused UST (Olympus-NDT, V309-SU) was employed to collect PA waves, with a central frequency of 5 MHz, active area of 13 mm, and fractional bandwidth of 70%. A 45 deg acoustic reflector (F102, Olympus NDT) was placed on the transducer body. 600 A-lines were obtained by continuously moving the UST in a circle around the sample for a scan time of 12 s, with each A-line containing 1024 samples. The UST was driven by the stepper motor (SM), which was controlled by the computer. The collected A-lines were amplified by a 48 decibels (dB) low signal noise amplifier (mini-circuits, ZFL-500LN-BNC), followed by being stored inside the computer (Intel Xeon, 3.7 GHz 64-bit processor, 16 GB RAM) via a DAQ card (Spectrum, M2i.4932-Exp). The DAQ was synchronized with the laser irradiation by the functional generator. Sprague Dawley rats (weighing 90±5  grams) were obtained from InVivos Pte. Ltd., Singapore for experiments. *In vivo* imaging was performed with one rat at a time, and the pre-imaging operations were the same for all rats. Before the imaging, each rat was anesthetized by a combination of ketamine (100  mg/mL) and xylazine (20  mg/mL), followed by hair removal by employing depilatory cream and the utilization of ocular gel on both eyes. A layer of ultrasound gel was applied to the rat scalp for better coupling. During the imaging process, 0.75% isoflurane and 1.0  L/min oxygen were continuously supplied to keep the rat anesthetized. After the experiments, the rat was euthanized by the intraperitoneal administration of Valabarb (sodium pentobarbitone 300  mg/mL). All the animal experiments were performed under the guidelines of the Institutional Animal Care and Use Committee, Nanyang Technological University, Singapore (Protocol No.: A0331).

### 2.4 Development of a Mobile-Platform-Based Algorithm

Python 3.9.5 was used for developing an Android-based application due to its several merits. Compared with another program, Android Studio, which is also commonly used for creating Android-based applications, Python is an interpreted language and can be used cross-platform. Many open-source libraries are available in Python, allowing users to flexibly call commands to accomplish certain tasks. Besides, users can call the functions in other languages like C and C++ to run the command more effectively since Python is extensible. In addition, to further improve the efficiency of the mobile-platform algorithm, we can employ Fourier-based or deep-learning-based reconstruction algorithms in the future, which can be more easily achieved in Python. A cross-platform Python framework, Kivy, was employed here for the development of the application. The codebase developed in this article mainly targets the Android system, though it can extensively target Linux, iOS, and Windows.

**Fig. 2:**
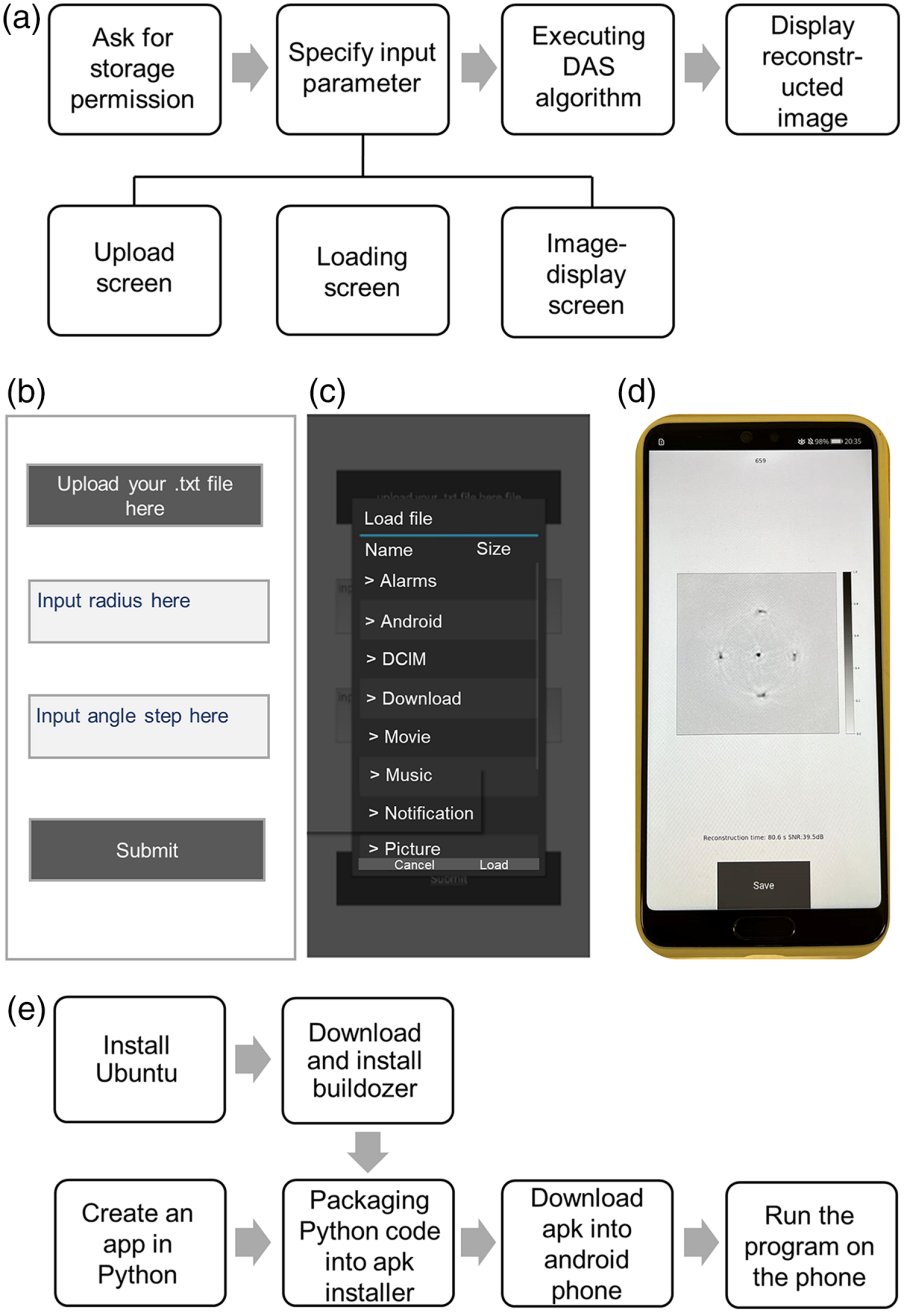
(a) How to develop Android app in Python via Kivy. (b) Upload screen. (c) Popup for uploading raw data to the application. (d) Image-display screen on the Android phone (Huawei P20), which showed reconstructed image, reconstruction time, and the SNR value. (e) How to transfer Python code to mobile platform.

The process of developing the application was illustrated in [Fig. 2(a)]. Storage permission was first asked for when opening the application, followed by uploading the raw data stored on phones. The upload screen and the popup for uploading files were shown in Figs. 2(b) and 2(c), respectively. Two parameters, including the radius and angle step, were provided by users in the upload screen. During the image-forming process, the loading screen was shown as the transition screen. After a while, the beamforming process finished and the image-display screen was exhibited, where the reconstructed image and output parameters were displayed [Fig. 2(d)]. The output parameters contained the running time of the image-forming process and the signal-to-noise ratio (SNR) value of the image, which were shown at the bottom of screen. The reconstruction algorithm used here was a simple DAS beamformer, which defined the reconstructed image size as 250×250  pixels (0.1  mm/pixel). The pixel size was smaller than both the spatial resolution values of the PLD-PAT system, which utilized a 5 MHz UST (resolution is ∼180  μm), and the Nd:YAG-PAT system, which employed a 2.25-MHz UST (resolution is ∼380  μm). In addition, optimization of the application was implemented by using effective functions, such as Enumerate and improving loading animation, aiming at shortening the time taken for reconstruction.

Although Kivy is cross-platform, packing the Python code into an executable program for the mobile platform is necessary. To run Python code on an Android phone, the code should be packaged into apk installer. There are several ways to finish packaging, including packaging via Python-for-Android (p4a), Kivy launcher, or Buildozer. In this work, we utilized Buildozer to package the Python code file into apk file since it was easily realized due to its integrated framework and can solve the dependencies of deploying to different platforms very well. In addition, while Buildozer debugging builds, the prerequisites were downloaded automatically, like p4a, Android SDK, and NDK, which indicated that Buildozer can further encapsulate p4a. However, Buildozer only compiled with Linux and macOS, and Windows is not supported. Before downloading Buildozer, a virtual machine or dual system was required to be installed for using Ubuntu, which was a Linux distribution based on Debian. After Buildozer was downloaded, an Android build could be initiated, leading to an apk file being created. By downloading apk into an Android phone, we can run the program on the phone. The whole process of transferring Python code into an executable mobile program was illustrated in Fig. 2(e).

### 2.5 Image Quality Assessment

Several image quality matrices were used to assess the reconstructed image quality. First, SNR was calculated after beamforming and normalization, using the following equations:

$$\mathrm{SNR}=20\;\log_{10}(\frac{\mu_{i}}{\sigma_{0}}),$$

where $\mu_{i}$ is the mean of the top ten signal amplitudes within a region of interest (ROI), and $\sigma_{0}$ is the standard deviation of noise amplitudes within ROI outside the photoacoustic targets. After reconstruction, the SNR value of the resulting image would be displayed at the bottom of the phone screen, expressed in dB. However, the absence of an upper limit in the measurement of SNR could create difficulties in the interpretation and comparison of results.^45^ Thus, two additional metrices were employed to evaluate image quality: peak SNR (PSNR), and structural similarity (SSIM) index. PSNR measures the ratio between the maximum possible value of an image and the amount of noise present in the image, expressed in dB. Higher PSNR values indicate better image quality. SSIM is another metric that measures the SSIM between original and reconstructed images, taking into account luminance, contrast, and structure. SSIM values range from 0 to 1, where a value of 1 indicates that the two images are identical in terms of structure and content. Like PSNR, higher values of SSIM indicate better image quality. Both PSNR and SSIM are full reference quality metrics, which compare the reconstructed image against a reference image without distortion. However, obtaining a reconstructed image without distortion in experimental datasets can be challenging. To evaluate the application’s performance, we used both simulated and experimental datasets and downsampled the data to observe how the reconstruction time changed with decreasing dataset size. The image reconstructed from the original dataset was considered as the reference image for comparison with the image reconstructed from the downsampled dataset. The following equations were used to calculate the PSNR and SSIM values:

$$\mathrm{PSNR}=10\;\log_{10}(\frac{{\text{peakval}}^{2}}{\mathrm{MSE}}),\;\text{where }\mathrm{MSE}=\frac{\sum \sum {\left(I-K\right)}^{2}}{m*n},$$

$$\mathrm{SSIM}=\;\frac{\left(2\mu_{x}\mu_{y}+C_{1})(2\sigma_{xy}+C_{2}\right)}{\left(\mu_{x}^{2}+\mu_{y}^{2}+C_{1})(\sigma_{x}^{2}+\sigma_{y}^{2}+C_{2}\right)},$$

where peakval is the maximum value of the reference image. MSE is the mean squared error between the reference image and reconstructed image, which computes the average of the squared difference between the pixel values of the reference and reconstructed images. m and n are the height and width of the images, respectively. x and y are the reference and reconstructed images and $\mu_{x}$ and $\mu_{y}$ are the means for x and y, respectively. $\sigma_{x}$ and $\sigma_{y}$ are the standard deviation of x and y, respectively. $\sigma_{xy}$ is the cross covariance of x and y.

## 3 Results and Discussion

### 3.1 Image Reconstruction on Mobile Platform

**Fig. 3:**
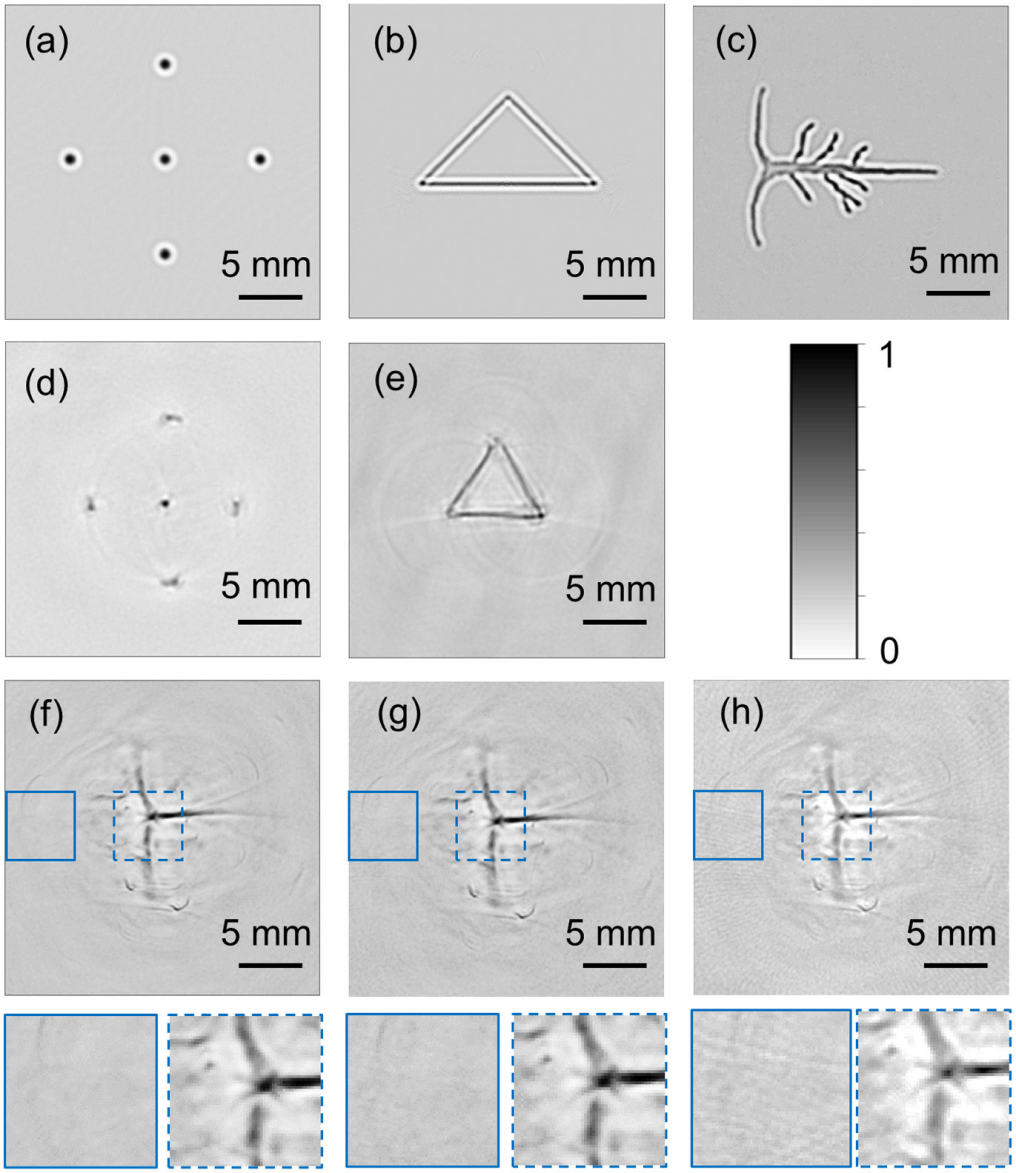
Reconstructed PAT images displayed on the mobile platform: images formed from (a) numerical point source data, (b) numerical triangle data, (c) numerical brain vessel data, (d) experimental point phantom data, and (e) experimental triangular phantom data. (f) *In vivo* brain vessel data, which were captured with 600 steps. (g) *In vivo* brain vessel data, which were downsampled to 300 steps. (h) *In vivo* brain vessel data, which were downsampled to 200 steps. The background region and a part of photoacoustic region (marked by blue solid box and blue dashed box) are shown in the bottom row.

**Fig. 4:**
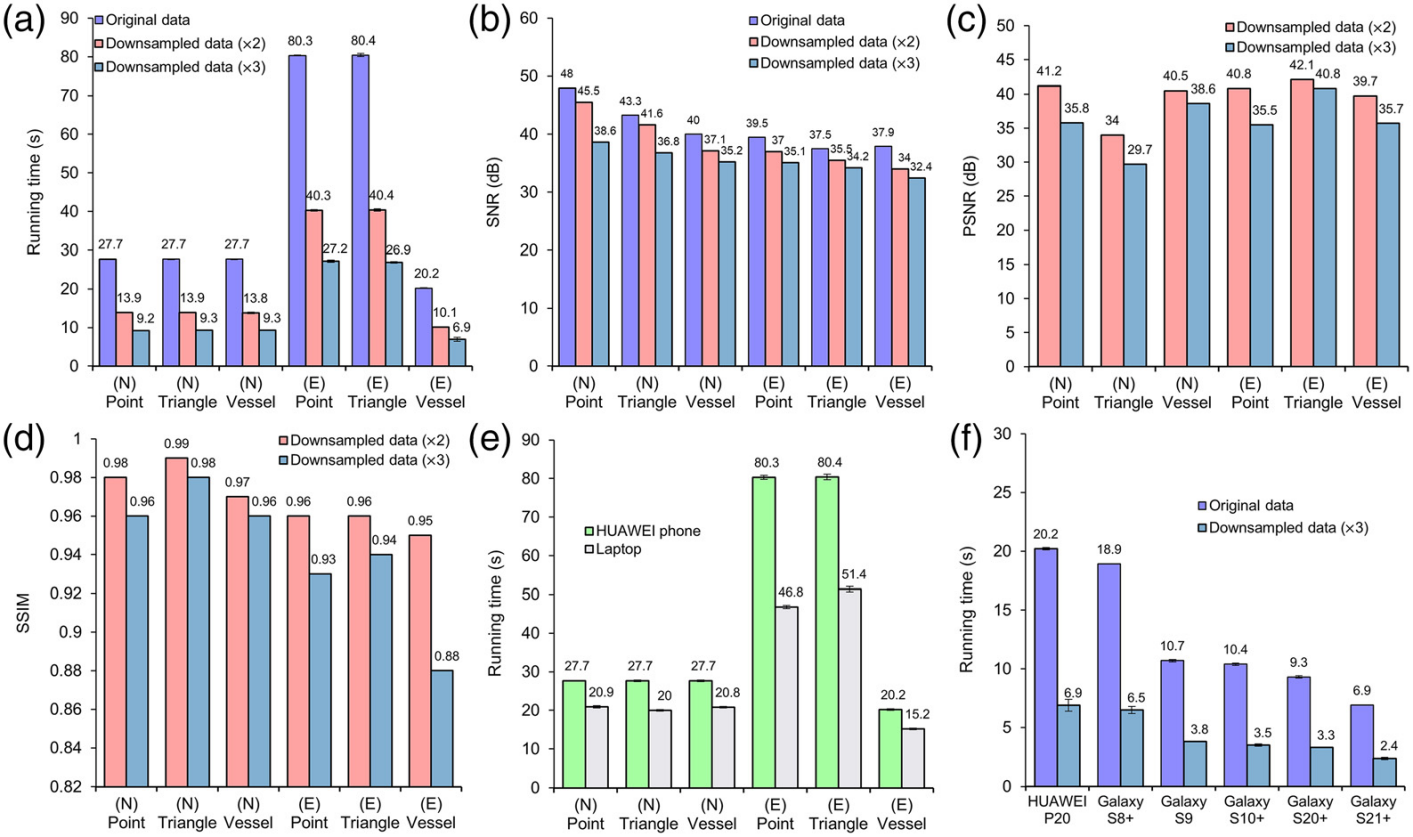
(a) Reconstructed time comparison among different datasets. (b) SNR comparison among different reconstructed images formed from different datasets. (c) PSNR comparison between images from twofold downsampled and threefold downsampled datasets; the images from original datasets were taken as reference images. (d) SSIM comparison between images from twofold downsampled and threefold downsampled datasets; the images from original datasets were taken as reference images. (e) Comparison of beamforming time on the mobile platform among different image-forming process. N, numerical dataset; E, experimental dataset. (f) Comparison of DAS image formation time on different mobile platforms; the data used here was *in vivo* original and downsampled data.

Figure 3 depicts the reconstructed images of both numerical and experimental datasets on Huawei P20 using the DAS algorithm. The SNR values of Figs. 3(a)–3(h) was displayed in Fig. 4(b). The reconstructed images of the numerical phantom are shown in Figs. 3(a)–3(c), in which the SNR values are 48, 43.3, and 40 dB, respectively. The structures of the targets have been properly reconstructed, which were well matched with the ideal condition as shown in Figs. 1(c)–1(e).

Although more A-lines were captured for experimental phantom imaging, the PA images formed from the phantom experimental dataset showed poorer quality compared with the PA images formed from the numerical dataset. This could be due to the noise added in the numerical datasets being lower than the actual experimental noise. The SNR values of Figs. 3(d) and 3(e) were 39.5 and 37.5 dB, respectively, around 8 dB lower than that of Figs. 3(a) and 3(b). The point targets were not well reconstructed, especially for the points that were far from the scanning center. This is expected, as the spatially variant tangential resolution due to the large aperture size of the detector used for experimental dataset is well known.^29^^,^^42^ As for Fig. 3(e), the structures of the triangle targets were reconstructed properly though the endpoints was blurred and the background noise was higher relative to Fig. 3(b). Using PLD-PAT system, rat brain was imaged. *In vivo* PA signals were captured at 600 uniformly distributed positions and 1024 samples were acquired in each position, resulting in a data size of 600×1024. The reconstructed images of the brain vessels were displayed in Fig. 3(f), with SNR of 37 dB. Both the transverse sinus and sagittal sinus were clearly observed, though some noises existed in the background.

To quantitatively evaluate the influence of data size on reconstruction image quality and time, six datasets were downsampled two times and three times in the number of positions around the circle. The reconstruction time for the original datasets and downsampled datasets was tested by five runs. Figure 4(a) shows the mean reconstruction time and standard deviation for each dataset, whereas Fig. 4(b) shows the corresponding SNR values of the reconstructed images. It can be found from Fig. 4(a) that for datasets of the same size (number of A-lines x samples in each A-line), irrespective of whether it is simulated or experimental data and what type of simulated data, the time for PAT image reconstruction was similar. Such relationship can be more easily seen in Table 1. Here, the reconstruction time was the average reconstruction time used for the same size of datasets. When the original dataset was downsampled twofold, the reconstruction time was reduced by approximately half, whereas the SNR value was only decreased by <4  dB. Considering the limitation of only taking SNR as image quality metric, PSNR and SSIM were also employed to assess the quality of images. As shown in Fig. 4(c), the PSNR values for all twofold downsampled datasets were higher than 40 dB, except for *in vivo* data, which was still ∼40  dB (39.7 dB). This reveals that the difference between the image generated from original datasets and twofold downsampled datasets was relatively low in terms of the maximum image pixel values and the noise levels. Also, the SSIM values of the reconstructed images from the downsampled datasets were all not <0.95. A high degree of similarity between the reconstructed images and reference images was thus demonstrated. Minimum distortions of the image reconstructed from twofold downsampled datasets can be further verified by the PA image reconstructed from two-fold downsampled *in vivo* data [Fig. 3(g)]. A part of background region and a part of photoacoustic target region were enlarged (marked by blue solid box and blue dashed box), suggesting that the structures of the photoacoustic target were still properly reconstructed, and the background was clean with not much noise perceptible. The quantitative and qualitative results demonstrate that the twofold downsampling process significantly reduced computation time without significantly degrading the image quality.

**Table 1:** Relationship between the size of dataset (no. of A-lines × samples in each A-line) and reconstruction time.

	Original dataset	Twofold downsampled dataset	Threefold downsampled dataset
Phantom data size	2400 × 1024	1200 × 1024	800 × 1024
Reconstruction time (s)	80.3	40.3	27.1
*In vivo* data size	600 × 1024	300 × 1024	200 × 1024
Reconstruction time (s)	20.2	10.1	6.9
Simulated data size	800 × 1500	400 × 1500	266 × 1500
Reconstruction time (s)	27.7	13.9	9.3

The reconstruction time could further be reduced by the three-fold downsampling operation, which led to about three times decrease in computation time. However, the images formed from threefold downsampled data show visible degradation (streak artifacts), as the SNR value of these images dropped compared to the images formed from original data. Streak artifacts are quite common when the number of A-lines used for reconstruction is low. The degradation can be observed from PSNR and SSIM values. The PSNR of the images formed from threefold downsampled datasets were around 35 dB, except for numerical triangle dataset (29.7 dB) and numerical brain vessel dataset (38.6 dB). The difference between the images from threefold downsampled datasets and original datasets became more pronounced, implying a loss of information during the reconstruction process (due to low number of A-lines). Similarly, the SSIM of images declined to around 0.96 for simulated datasets except for numerical triangle dataset, around 0.93 for experimental phantom dataset, and even lower, 0.88 for *in vivo* dataset, which implied a lower degree of similarity between the reconstructed image and the reference image. However, the SSIM values higher than 85% still demonstrate the proper reconstruction of target structure, though some degradation was present (more pronounced in the background areas). It is worth noting that the SSIM value for the image from numerical triangle dataset is highest among the images formed from threefold downsampled datasets, though the PSNR value of that is the lowest. Such divergence could be due to the fact that the image had a high SSIM with the reference image though it has undergone some form of compression that has reduced the pixel values, resulting in a lower dynamic range and a lower PSNR. But the image still retained the essential structural features of the reference image. Figure 3(h) showed the reconstructed image from three-fold downsampled *in vivo* data in which the background artifact (streak artifact) was notably higher than Fig. 3(f), leading to an SNR decrease of more than 5 dB. But three-fold downsampling process still led to the PA images with comparable target structure to that of the PA images obtained from the original datasets, which was consistent with the SSIM value of the image.

These findings suggested that there was a tradeoff between image quality and reconstruction time, as larger-sized datasets led to images with higher quality but longer reconstruction time, whereas smaller-sized datasets resulted in lower-quality reconstructed images but reduced reconstruction time. Limited by the size of the screen and screen resolution, smartphones cannot display high-resolution images as clearly as desktops/workstations/laptops, indicating that the phone application can focus more on the time consumed for the image-forming process instead of image quality. Considering the influence of downsampling on reconstruction time and image quality, two-fold downsampling led to much less time spent on beamforming but still maintained the image quality without much loss, offering a balance between speed and quality. An additional step was done to verify that transferring the Python file into apk file would not influence the reconstructed image quality. We employed the same DAS algorithm and the same raw dataset in laptop Python to obtain the reconstructed images, for which the SNR values were the same as that of images reconstructed on mobile platforms.

### 3.2 Reconstruction Time Comparison Between Mobile Platforms and Laptop

Figure 4(c) demonstrates the reconstruction time difference between the laptop and mobile platforms (Huawei P20) among different samples. The laptop used here was Lenovo Thinkbook 15, possessing 24 GB DDR4 memory and a processor of 10’th Gen Intel^®^Core™i7. To evaluate the stability of the application, five runs have been tested on six datasets on both laptop and Huawei P20. The mean time and standard deviation were calculated for the reconstruction time. The datasets used here were maintained in their original size without any downsampling performed. As for the same dataset, the time taken for reconstruction in Huawei P20 was significantly higher than that on the laptop. Around 7-s differences were observed between the image reconstruction time on laptop and mobile platforms for all numerical datasets (800 steps × 1500 samples). For larger-size datasets (2400 steps × 1024 samples, for experimental phantom datasets), the time spent on reconstruction showed a huge difference between laptop and mobile platforms. Around 50 s was required for the laptop to form the PAT images, and the mobile application took an additional 30 s to fulfill the same beamforming process. The significant difference in the reconstruction time in the two platforms was mostly owing to the relatively less advanced processor of Huawei P20, which was released four years ago. *In vivo* brain vessel dataset comprised fewer DAQ points (600 points), resulting in significantly less time for DAS. Without performing downsampling on the raw dataset, there was about a 5-s reconstruction time difference between the two platforms. These results indicated that the mobile application possesses no significant running time difference from the laptop when performing the DAS for the small-sized datasets, whereas a significant time difference would be there between the two platforms for the large-sized datasets. Performing downsampling to the dataset can accelerate the image reconstruction, and the reconstructed image showed not many differences from the image reconstructed from the original large dataset. In addition, the standard deviation of five runs on the mobile platforms was lower than 1 s, suggesting the stability of the application.

### 3.3 Reconstruction Time Between Different Mobile Platforms

Mobile phone performance varies significantly with different specifications, including central processing unit (CPU), GPU, and internal memory size. To measure the extent to which different specifications affect reconstruction time and verify that the application can be universally applied on Android platforms, we compared the running time on six different Android phones, as shown in Fig. 4(d). Their specification is presented in Table 2, and Samsung Galaxy S21+ is the most recent Android phone that was tested. *In vivo* brain vessel dataset was used here, with both the original (600-step) and downsampled (200-step) datasets. The first developed version of the application required 127.7±8.4 seconds to accomplish the image-forming process for the 600-step *in vivo* brain dataset on a Samsung Galaxy S9 phone. Owing to performing optimization operation including importing only the required modules of a library, employing functions with short execution time, and using smoother loading animation, the reconstruction time was reduced to 10.7±0.0  s.

**Table 2:** Specification of seven Android mobile platforms.

Specifications	Huawei P20	Samsung Galaxy S8+	Samsung Galaxy S9	Samsung Galaxy S10+	Samsung Galaxy S20+	Samsung Galaxy S21+
Released year	2018	2017	2018	2019	2020	2021
RAM	6 GB	4 GB	4 GB	8 GB	8 GB	8 GB
Internal memory	128 GB	64 GB	64 GB	128 GB	128 GB	256 GB
CPU	Octa-core (4 × 2.4 GHz Cortex-A73)	Octa-core (4 × 2.3 GHz and 4 × 1.7 GHz)	Octa-core (4 × 2.7 GHz Mongoose M3 and 4 × 1.8 GHz Cortex-A55)	Octa-core (2 × 2.73 GHz Mongoose M4 and 2 × 2.31 GHz Cortex-A75 and 4 × 1.95 GHz Cortex-A55)	Octa-core (2 × 2.73 GHz Mongoose M5 and 2 × 2.50 GHz Cortex-A76 and 4 × 2.0 GHz Cortex-A55)	Octa-core (1 × 2.9 GHz Cortex-X1 and 3 × 2.80 GHz Cortex-A78 and 4 × 2.2 GHz Cortex-A55)
GPU	Mail-G72 MP12	Mali-G71 MP20	Mali-G72 MP18	Mali-G76 MP12	Mali-G77 MP11	Mali-G78 MP14

With similar release year and configurations, Huawei P20 and Samsung Galaxy S8+ completed image forming process within similar time for both datasets. 6.9 s and 20.2 s were needed for Huawei P20 to perform image reconstruction in 200-step and 600-step brain vessel data, respectively. Similarly, 6.5 s and 18.9 s were required for Samsung Galaxy S8+ to finish the beamforming process in both reduced-sized and original datasets. For the Samsung Galaxy series of smartphones with improved specifications, the reconstruction required less and less running time. Compared to Huawei P20, the newly released Samsung Galaxy S21+ possessed an advanced processor, with >60% decrease in time spent on the image-forming process. 2.4 s and 6.9 s were required for the reconstruction of downsampled dataset and the original dataset, respectively. The less running time revealed the possibility that the advanced mobile system owned similar or even superior performance in the image-forming process compared to the laptop.

In this work, the reconstructed image size was fixed at 250×250  pixels (0.1  mm/pixel), which means that only the photoacoustic targets within a region of 2.5×2.5  cm can be reconstructed. However, the image size can be adjusted arbitrarily by modifying the parameters that control its height and width in the Python scripts. As for the dataset size, there is no limit as long as it does not exceed the maximum memory capacity of the phone. The development of an Android-based application for photoacoustic image reconstruction is expected to contribute significantly to the advancement of portable phone-based photoacoustic imaging systems. These systems have the potential to improve medical diagnostics and make healthcare more accessible to underserved populations. However, building such systems has been a challenge due to the difficulty of integrating high energy light sources or the ultrasound detectors within the phone. Advancement of LED light sources could accelerate the development of mobile PA imaging system. Mobile based ultrasound imaging systems are already developed. Potentially one can modify such a system to get the raw channel ultrasound radio frequency (RF) data (not a trivial task though), as already beamformed RF data will not be useful for PA imaging. On the other hand, controlling the hardware of an external laser (or other potential light source) from a phone, DAQ, digitization and transfer into a mobile phone might be possible but has not been demonstrated yet. So, there are few different paths through which it might be possible in the future to develop the full PAT imaging system on a mobile. Although on the image acquisition side not much work has been done for a mobile-based PAT system, it might be feasible to do so soon. Our future work will focus on not only the mobile based reconstruction but also developing mobile based PAT imaging system.

## 4 Conclusion

In this work, we use the potential of mobile platforms for PAT image reconstruction. Simulated datasets and experimental datasets were both used to evaluate its performance. The results indicate that the images shown on mobile phones were reconstructed well, with all the SNR values of reconstructed images >30  dB. Significantly longer image reconstruction time was needed for mobile applications, especially for large-sized datasets. Two-fold downsampling operation could be an alternative for lowering the time spent on beamforming but still maintaining the image quality without much loss. Different types of phones have been employed to compare the application performance. With the newly developed phones that possess powerful GPU and CPU, the required time for image forming can possibly be as close as to the time needed for reconstruction on the laptop. Although demonstrated by the dataset from the PAT system with a single-element transducer, the proposed application is sufficiently broad and general that it should directly be applied to the dataset from the PAT system with an array-based transducer, like a circular-array transducer.

## Code Availability

The source code of the developed app is available at GitHub: https://github.com/DeeplearningBILAB/Android-mobile-platform-based-image-reconstruction-for-photoacoustic-tomography. The apk file can be downloaded from Open Science Framework (OSF).^46^
